# Insights from the Molecular modeling, docking analysis of illicit drugs and Bomb Compounds with Honey Bee Odorant Binding Proteins (OBPs)

**DOI:** 10.6026/97320630014219

**Published:** 2018-05-31

**Authors:** Kulanthaivel Langeswaran, Jeyakanthan Jeyaraman, Richard Mariadasse, Saravanan Soorangkattan

**Affiliations:** 1 Department of Bioinformatics, Alagappa University, Karaikudi-630 003; 2 Department of Botany, Alagappa University, Karaikudi - 630 003

**Keywords:** Biosensor, Docking, DFT, Honey Bee, Illicit drugs, OBPs, olfactory sensory, Phylogenetic tree

## Abstract

Analysis of honeybee PBPs is of interest in the development of Biosensor applications. We described the predicted binding of 19 such compounds with 43-honey bee OBPs using molecular modeling, docking and phylogenetic analysis. Therefore, training the honeybees using preferred compounds formulate the bees to identify the illicit drugs and bomb compounds. Consequently, high docking score produced complex such OBP16-N-Phenyl-2-Napthalamine (-12.25k/mol), 3BJH-Crack Cocaine (-11.75k/mol), OBP10-Methadone (- 11.71k/mol), 1TUJ-Dronobinal Cannabis (-11.66k/mol), OBP13-Plasticizer (-11.27k/mol) and OBP24-Ecstasy (-10.89 k/mol) can be used to identify the compounds using biosensor application. The chemical reaction of the compounds for olfactory sensory was analyzed using DFT (Density Functional Theory) studies. Some of these compounds show high binding OBPs across distant phylogeny.

## Background

In 2013, it was estimated that 24.6 million people around the age group of 12, which is approximately 9.4% of the population using Illicit drugs in America. It is also found that 5% (i.e. 230 million) of world's adult population is consuming Illicit drugs [1]. The most commonly available drugs are cannabis, heroin, opium, methadone, amphetamine, cocaine and hashish etc. Drug addiction is a vital problem in their families and it directly gives way to financial crises of family income and health issues [2]. Also, these illicit drugs are directly affecting the health of the person and gives approximately 0.2 million deaths per year, in which, heroin and cocaine are major causative agents [1].

Sniffer dogs have the ability to smell and detect the crime, but their ability threshold of smell is lesser than commercially available analytics [3, 4]. Moreover, in terms of disadvantage, the cost and training duration is huge in the short term and the biased activities of trainer, can lead the dogs to perform positive or negative response [5]. Recently, the US government announced, cannabis drug is legalized in the country therefore; detection by sniffer dogs cannot be taken as evidence for the probe of crime [6, 7, 8]. Therefore, the alternative solution using insects can be a better idea of the identification of illicit drugs [3]. The high smell sensing nature and learning capability, insect is an alternative biosensor application.

One among the insect is a honeybee. There are several types of honeybees present globally but Apis mellifera (western honey bee) and Apis cerena (Asian honey bee) are significant [9] among them. Honeybee has high sensing capacity and detect the odor compounds in the floating air and find the place where the source of food available [10]. Honeybees have more than 177 odorant binding genes that are responsible for the detection of volatile compounds [11]. The antennae of honeybee are more sensitive and useful for detecting the volatile compounds [12]. Recent research suggesting that, training the honeybees can detect and locate the bomb compound TATP (triacetone triperoxide); therefore, the explosive material can be easily identified. [13]

Honeybee's chemical communication occurs via the acid watersoluble proteins that recognized the airborne hydrophobic odorant compound to olfactory sensing systems. These proteins can be classified as Odorant binding protein (OBPs), Pheromone Binding Protein (PBPs) and chemosensory proteins (CSPs). In which, OBPs are commonly used to recognize various odorant compounds binding specificity and induce the first step signal to the olfactory sense. PBPs constituent in general, male bees detect the sex pheromones released by the queen bees. CSPs proteins recognized the chemical compound for the communication of insects.

OBPs of honeybee classified three Antenna Specific Proteins (ASPs) such as ASP1, ASP2 and ASP3 diverse in the antenna and functioning differently. ASP1 protein belongs to pheromone binding protein (PBP) since it binds to detect the 9-keto-2(E)- decenoic acid and 9-hydroxy-2 (E)-decenoicacid of queen pheromones. ASP2 proteins consist of diverse sequence variation with PBPs therefore; pheromones did not bind with ASP2 protein considered as OBPs. The ASP3 proteins are highly homologous with the CBPs groups that classified under CBPs protein. Among these proteins, ASP2 is well-characterized OBPs proteins for binding affinities of ligand and volatile compounds. Homologous of ASP2 protein with other OBP protein structures may depict the functional concurrence of ligand binding affinity for olfactory sensory.

The rational approach towards the identification of elicit drugs and bomb compounds using honey bee Odorant binding proteins are most important phenomena for the identification of compounds [3]. The computational approaches are the most successive method for understanding the binding preferences and the chemical reaction of the biological function. In this study, we have performed phylogenetic tree, extensive docking and DFT studies to understand the binding mechanism and the mode of illicit drugs and bomb compounds interactions with OBPs of honeybee [14]. As consequent, honey bee and OBPs can be used in two different ways based on the binding preference and binding score [15]. If the binding preferences of illicit drugs and bomb compounds are high towards OBPs, it can train the honey bees to identify the source compounds, whereas, if the binding score (scoring functions are fast approximate mathematical methods used to predict the strength of the interaction (also referred to as binding affinity) between two molecules after they have been docked) is high, we can develop the molecular biosensor application using respective OBPs to detect the illicit drugs and bomb compounds. This method may pave the application towards the identification of illicit drugs and bomb compounds using honeybee Odorant Binding Protein.

## Methodology

### Compounds and Protein collection from Databases

The easily available nineteen illicit drugs and bomb compounds were obtained from the literature studies (R, S) and the 3- Dimentional structures of the compounds were retrieved from the PubChem database [16] (Table 1) Nineteen illicit drugs drugs and bomb compounds collected from PubChem database). Threedimensional structure of 10 Odorant binding proteins (OBPs) and 33-odorant binding protein sequences of honeybee were retrieved from PDB and UniProt database [17]. Retrieved sequences were further used to construct the 3-D model using Swiss-model server [18] to understand the secondary structure elements and structural proteins. Totally, 43 OBP structures and 19 illicit drugs and bomb compounds were used for the further studies.

### Sequence, Secondary Structural element and phylogenetic tree analysis

All the 43 sequences of OBPs were used to perform the multiple sequence alignment using Clustal W [19] and the functional domain of the proteins was identified using CDD server [20] to understand the contribution of sensing nature. The phylogenetic tree analyses were performed using Mega 7 software [21] and JTT amino acid substitution model was used to generate the tree with the bootstrap value of 1000. One sequence from each clade was taken and their 3-D structure was superimposed to understand based on the secondary structure element. The structure based phylogenetic tree was constructed and the relation between the protein structures was analyzed [22].

### Ligand Preparation

3-D structures of all the illicit drugs and bomb compounds were prepared using LigPrep module implemented in the Schrodinger software suite, version 3.3 [23]. The implicit hydrogen was removed and appropriate hydrogen atoms were added to the structures for the minimization. The unwanted water molecules were removed. The expand protonation and tautomeric states at 7.0 ± 2.0 pH units were applied in order to generate the lowest energy structures of the illicit drugs and bomb compounds. The Partial atomic charges were computed using the OPLS_2005 force field [24].

### Protein Preparation

The retrieved 33 OBPs sequences were used to develop the 3-D model of protein using Swiss Model [18] and 10 structures taken from PDB database were considered as receptor molecules. The OBPs proteins were prepared and refined using protein preparation wizard implemented in the Schrodinger software suite [22]. The hydrogen atoms were consistently added to the protein structures with the pH 7.0±2.0 subsequently minimized with Optimized Potential for Liquid Simulation (OPLS-2005) all atom force field [23]. Energy minimization was performed to constraining the heavy atoms with the hydrogen torsion parameter turned off, to allow free rotation of hydrogen atoms. Restrained minimization was terminated until the maximum Root-mean-square deviation of non-hydrogen atoms reaches 0.3 Å. The proteins can use to predict the active site packet using Sitemap module [26] to generate the active site zone and the Grid [27] was generated to dock with volatile compounds [23].

### Docking studies

The Docking studies of 19 compounds with 43 OBP proteins were performed usingXP mode using Schrodinger software suite [23]. The active site residues of OBPs and their interactions were identified using Ligplot module. The 19 compounds preference is highly relying upon active site cavity and charged surface of the OBP proteins. The compounds and the 2D ligand interaction diagram will indicate the type of interactions with the key amino acid residues in the active site of OBP. Based on the Docking score and Binding free energy, the potential compounds will be detected and predict specific compound attracting the honeybee to find out the illicit drugs and bomb compounds [3].

### Molecular property analysis of proteins

The molecular electrostatic potential surface of the OBPs was carried out using PyMol Software (Schrodinger, LLC) [28]. The charged density of the proteins can prefer the compounds to bind to the active site of the proteins. Binding selectivity of the compounds highly depends upon the nature of the protein surface. The positive surface denoted by the blue color region and the red color region indicated negatively charged regions. The neutral region denoted by the white color (protein 6 (GAS6) and protein S (PROS1).

### DFT calculation

In the quantum, mechanical calculation, DFT calculates the molecular electronic features such as electron density and frontier molecular orbital (HOMO and LUMO) to predict the biological activity and molecular features of the compounds [29]. Geometry optimization was performed using a hybrid DFT approach at B3LYP (Becke's three-parameter exchange potential and the Lee- Yang-Parr correlation functional) with 6-31G* basis set. The Poisson-Boltzmann solver was used to calculate the energy in aqueous condition to simulate a physiological condition, which provides the information about the global and local indices of ligand molecules to their biological activity. The spatial distributions of electronic features in charge transfer mechanism are obtained from the HOMO and LUMO molecular orbitals. All DFT calculations were carried out using Jaguar, version 8.7 [29] to define the role of illicit drugs and bomb compounds.

## Results & Discussion

### Sequence, secondary Structure and phylogenetic tree analysis

**Figure 1:**
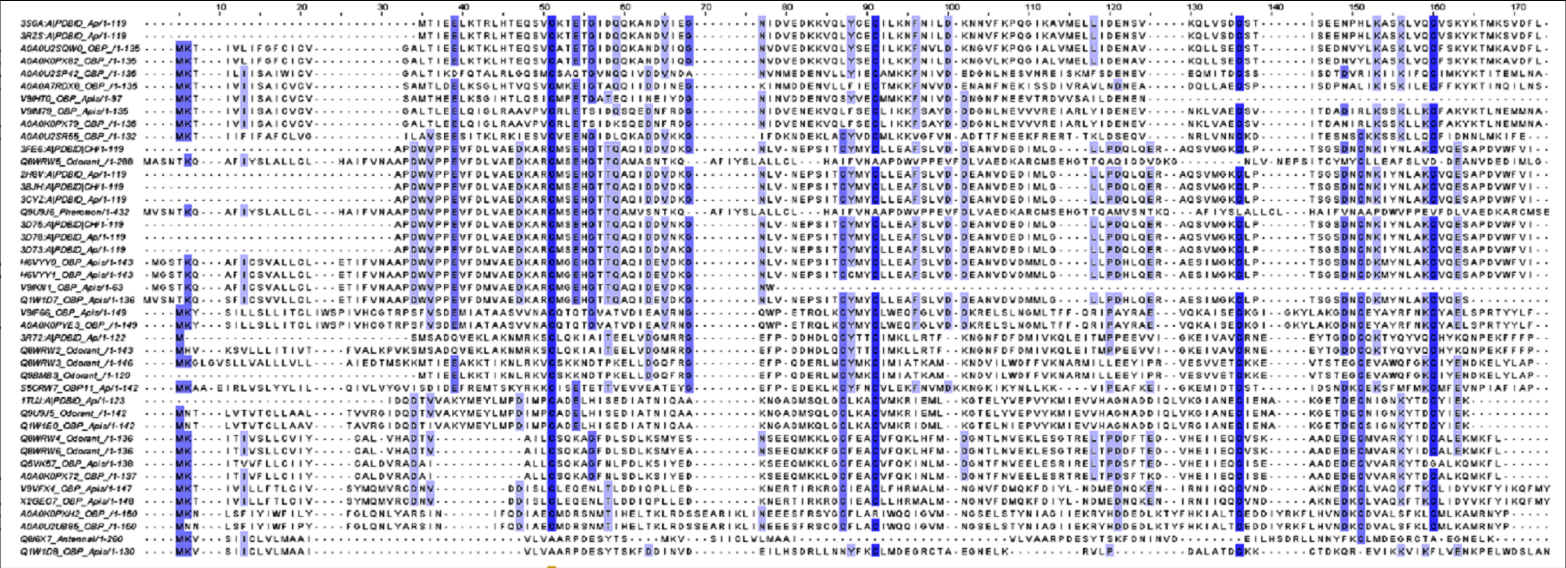
Multiple sequence alignment of 43 OBPs from honeybee. The residues shown in blue color depicts conserved residues within the group of organisms.

**Figure 2:**
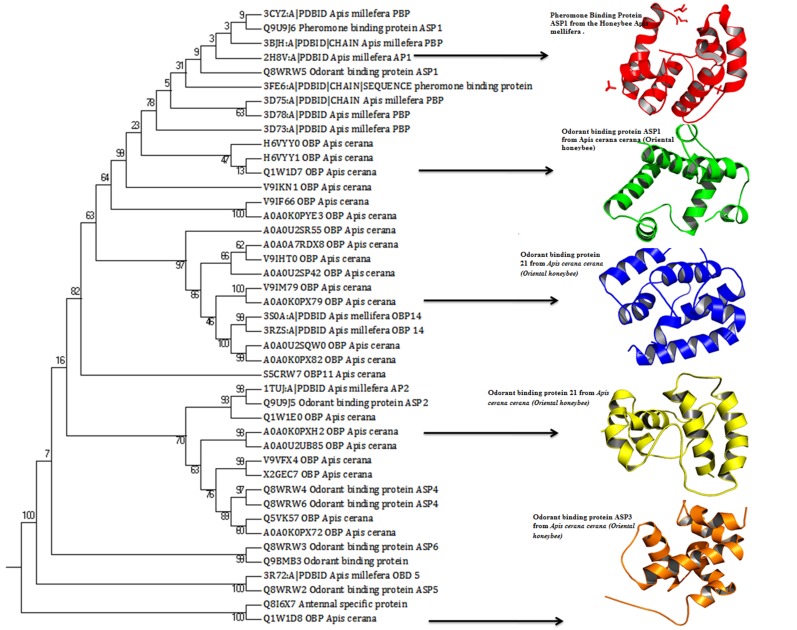
Structure based phylogenetic tree analysis of 43 OBPs using Mega 7 software.

**Figure 3:**
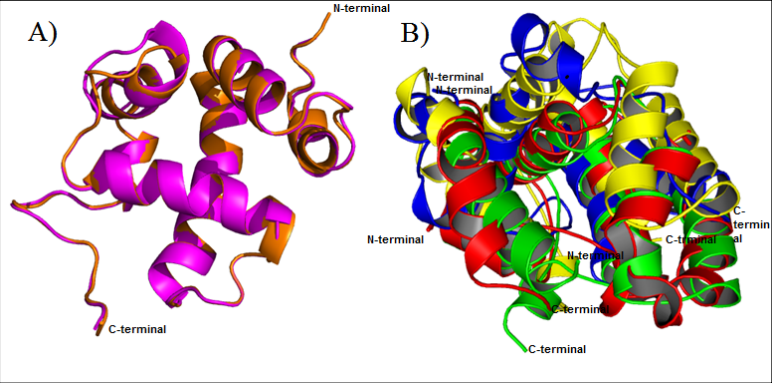
Superimposition of six OBPs taken from the phylogenetic tree. A) The two structures of OBPs from out group of phylogenetic tree (Pinkc color-ApisMillefera(model-Q8I6X7) and Orange color-Apis Cerana (Model-Q1W1D8). B) Superimposition of four OBPs from in-group of phylogenetic tree (Red-2H8V, Green-Model A0A0K0PXH2, Blue-Model A0A0K0PX79 and Yellow-Model Q1W1D7).

**Figure 4:**
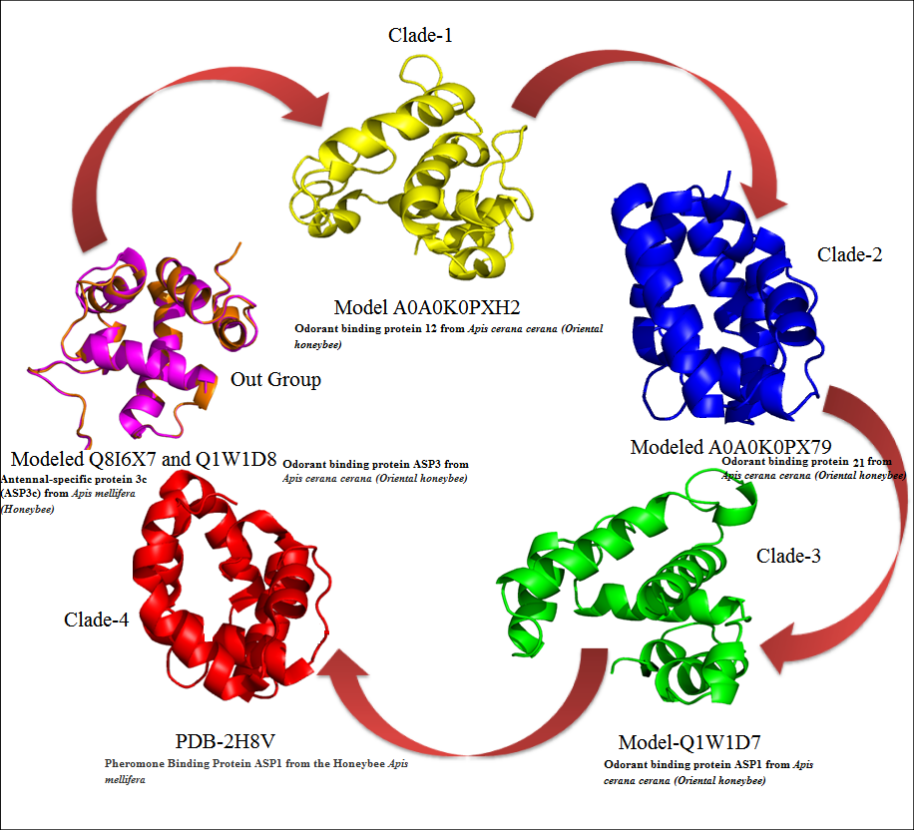
Structural evolutions of six OBPs taken from Phylogenetic tree.

The sequence analysis of 43 OBPs sand the secondary structural element were analyzed. The result enlightens all the OBPs sequences are similar in nature and consist of conserved and semi-conserved residues within the group of organisms. Cysteine residue falls highly conserved in all the sequences, whereas, Glycine, Glutamic acid, Aspartic acid, Valine, Lysine, Methionine, Glutamine, Threonine, and Asparagine amino acids found to have conserved within some OBPs (Figure 1). Cysteine residue in OBPs may contribute the protein stability and Lysine, Asparagine, Aspartic acid, Glutamic acid may contribute to the charged surface of the OBPs. Moreover, there is no conserved domain constituent in the all the OBPs sequences, therefore, the structural foldmay differ from one to other proteins. All the 43 OBPs consist of six or seven α-helices in the structure. Due to this, the active site pocket surface of the proteins may influence the binding affinity of the compounds. Depends upon the amino acid composition of proteins, the electrostatic surface and their based binding selectivity of compounds can differ. Moreover, the structural superimposed of 43 OBP proteins reveals that 26 proteins secondary structural elements were retained in the structural integrity and remaining16 proteins consist different folds of secondary structural elements. This difference in the structure leads to focus on the structural aspect to investigate the binding mode of illicit drugs and bomb compound and their related biological function. The structure based phylogenetic tree approach leads to understanding the structural similarity of OBPs and their electrostatic attribute to determine the binding specificity of compound (Figure 2). The relationship among the OBPs of ApisCerana and ApisMillifera organism was identified using phylogenetic tree analysis. The tree consists of three major clades and out-group of rooted tree depicting the ancestral lineage. The OBP16 and OBP23 proteins belong to Apis Cerana and Apis Millifera family of honeybee proteins are highly homologous in their sequences and the structure. The bootstrap value of phylogenetic tree explores the less noise with good quality of the tree. Moreover, superimposition of protein structures from each clade were depicts, structural folds of OBPs are highly similar and could find the difference in the binding cavity of the protein surface which determines the selectivity of compounds according to the binding sites (Figure 3). Apis Cerana and Apis Millifera has highly homologous in the sequences and the structural properties, therefore it can be a better model if we produced the any of one protein from two different organisms. The structural evolution of OBPs based on the phylogenetic tree shown in (Figure 4).

### Molecular electrostatic potential surface analysis

**Figure 5:**
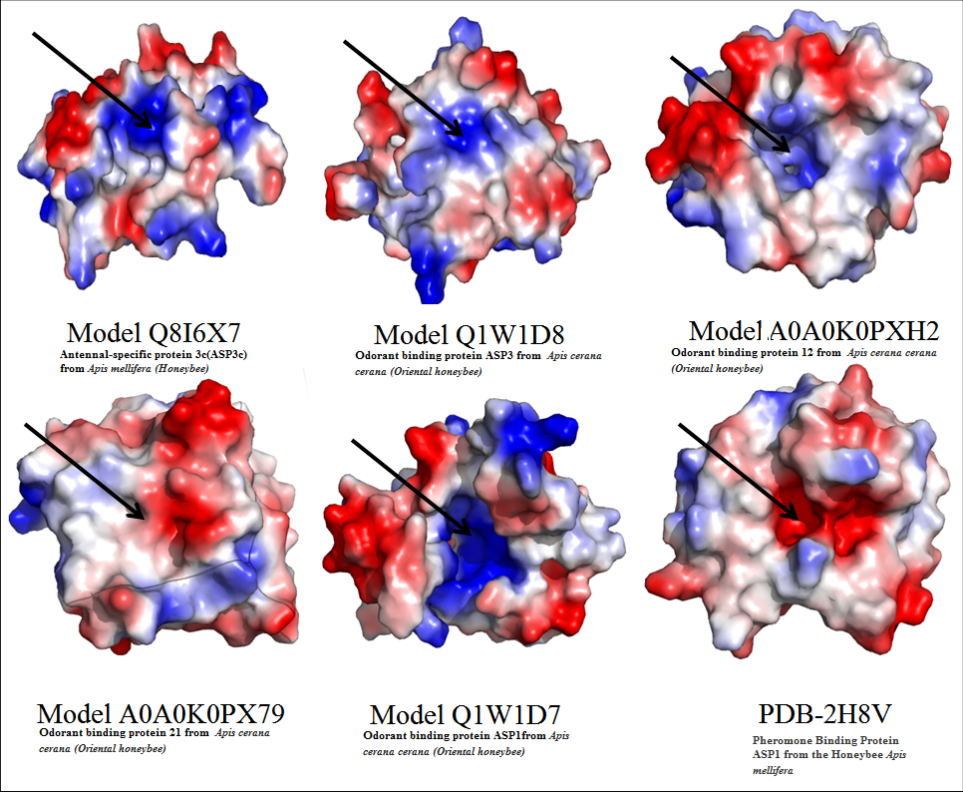
Electrostatic surfaces of six structures from phylogenetic tree depicting the charge variation in the structures.

The molecular electrostatic potential analysis was performed for each OBP from different clades of the phylogenetic tree to understand the contribution of the charged density of the proteins for the binding specificity of the compounds. Six proteins from different group of the organism were accounted and the electrostatic surface was analyzed. Interestingly, modeled OBP16 and OBP23 protein structures belong to Apismillifera and Apiscerana honey that consists of positive and neutral charged surfaces in organism depicts the similar binding cavities. In the case of Clade 1 and II, OBP4 and OBP2structurallyhomologous and the electrostatic surface showed that positive surface located in OBP4 protein whereas OBP2protein consists of negative charged surface. The modeled structure of OBP22 and 2H8V crystal structures from Clade 4 and 5 showed that modeled OBP22 protein structure consists of a positive surface whereas 2H8V protein consists of negatively charged in the active site pockets. Charged residues in the active site pocket of the proteins contributed the binding selectivity and affinity of the compounds. Also, amino acid substitutions in the active site pockets confer the differential electrostatic surface and binding cavities volume to the binding of compounds therefore, the proteins may have undergone the structural divergence and present in the different cladesphylo genetic tree. This may contribute the binding preference of the 19 illicit drugs and bomb compounds for binding selectivity according to amino acid substitutes and electrostatic surfaces. The electrostatic interactions of OBPs were shown in (Figure 5) to understand the charged surfaces of six proteins taken from the phylogenetic tree.

### Docking studies of illicit drugs and bomb compounds

**Figure 6:**
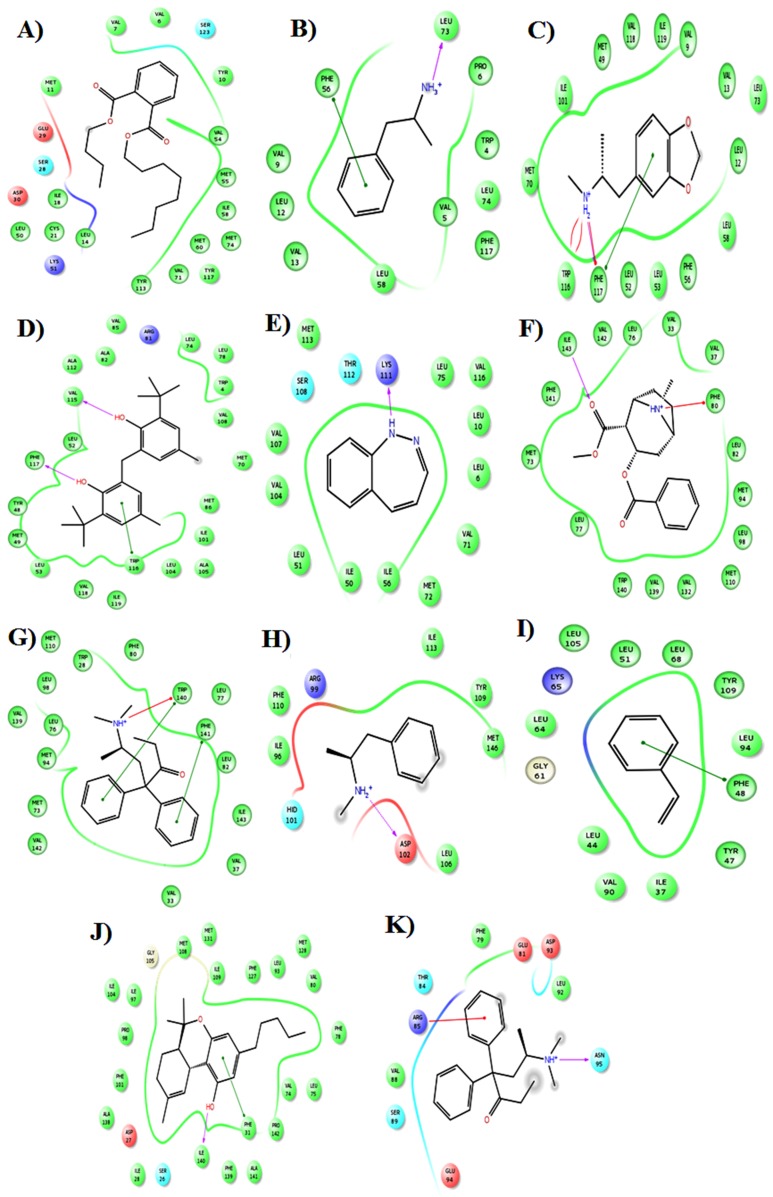
Interaction of Eleven Illicit drugs and bomb compounds with OBPs from honeybee

Docking studies were performed using all the 19 compounds with each 43 proteins to understand the binding specificity and the mode of interactions. This entire work is highly relied upon binding of illicit drugs and bomb compounds and not on neither docking score nor preferences (how many proteins prefer one compound) of the compound. Therefore, we fix criteria that docking score is more than -10.00 kcal/mol is considered as better docking score. Result enlightens that, all the compounds were not found to have a better binding score (Below -10.00 kcal/mol) and only 11 compounds show a high binding affinity with more than -10.00 kcal/mol with OBPs (Table 1). Docking of N-Phenyl-2-Napthalamine with modeled Q8WRW4 protein have a high docking score of -12.25k/mol. Likewise, 3BJH-Crack Cocaine (-11.75k/mol), H6BYY1-Methadone (-11.71k/mol), 1TUJDronobinal Cannabis (-11.66k/mol), S5CRW7-Plasticizer (- 11.27k/mol) and Q1W1E0- Ecstasy (-10.89 k/mol) shows the high docking score with the selective specificity of the compounds. Interestingly, the bomb compounds of N-Phenyl-2-Napthalamine (-12.25k/mol) shows the high docking score with OBPs followed by that, Crack cocaine (-11.75k/mol) shows the better docking score in the active site pocket of proteins. Moreover, different types of interactions like H-bond interaction, Pi-Pi interaction and ionic interaction found with the OBPs of the honeybee to favor the reactions. Compounds such as Amphetamine, methamphetamine and N-Phenyl-2-napthalamine are aminecontaining moiety therefore, it forms H-bond with negatively charged residues of the proteins [30]. Moreover, Binder Styrene Butadiene and methadone compounds consist of one or two aromatic rings in the structure therefore, it could not form any Hbond interactions rather it forms pi-pi stacking with respective proteins with high docking score. Most of the interactions were found to have charged amino acids such as Aspartic acids and positively charged amino acids Arginine in the active site pocket. Depends upon the interactions, the biological function of the honeybee detecting may vary per the compounds. The docking score of all the compounds with respective OBPs is shown in (Table 2). The interaction of residues of all the compounds and their mode of interactions depicted in the (Figure 6). High docking score prefers the compound to bind well in the active site pocket. Based on this study, we can use these proteins at molecular level biosensor application to detect or identify the illicit drugs and bomb compounds.

### Binding selectivity analysis of illicit drugs and bomb compounds using OBPs

**Figure 7:**
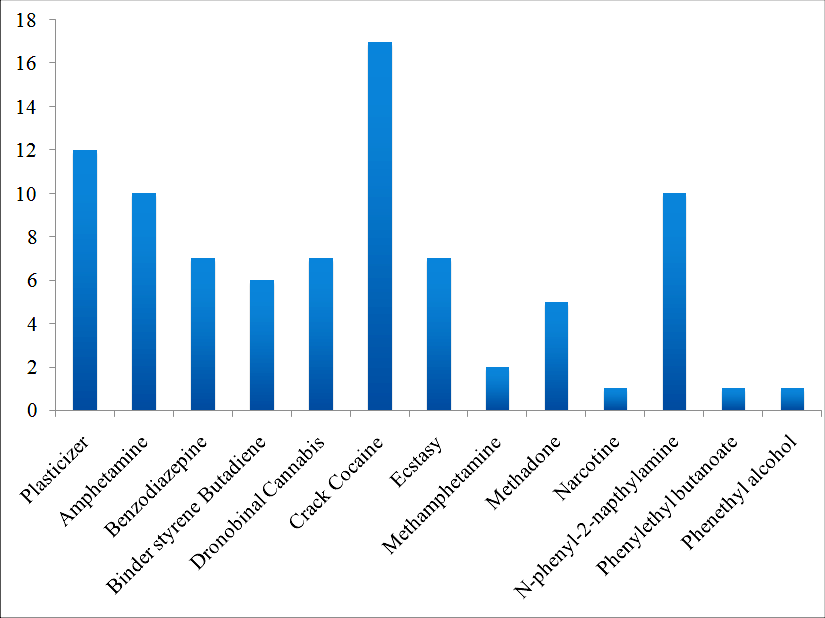
Binding selectivity of illicit drugs and bomb compounds with 43 OBPs. Top two docked compounds were accounted for the binding preference analysis.

Here we have analyzed the Binding preference of illicit drugs and bomb compounds with 43 OBPs (Details of 43 OBPs given in Table 3). Docking of 19 compounds with 43 OBPs, each protein may often prefer one compound; therefore, the probability of signaling mechanism in honeybee may induce the memory to identify the compounds. Hence, the training of those compounds with honeybee leads to identify the compounds where it is present. Among the 43 OBPs, several proteins highly binding prefer Crack Cocaine, Plasticizer, N-Phenyl-2-Napthalamine, Dronobinal Cannabis, Ecstasy, Benzodiazepine, Binder styrene Butadiene and Methadone predominantly in the active site of proteins. This binding nature can induce high sensing power of honeybee to memories and detect the compound in the respective source of food. Observing from this study, training of honeybee using those compounds can be easy to identify the bomb and illegal drugs. Because of high selectivity compounds toward the binding would be important for the sensing nature of honeybees. Figure 7 shows the binding selectivity of the illicit drugs and bomb compounds.

### DFT studies analysis

**Figure 8:**
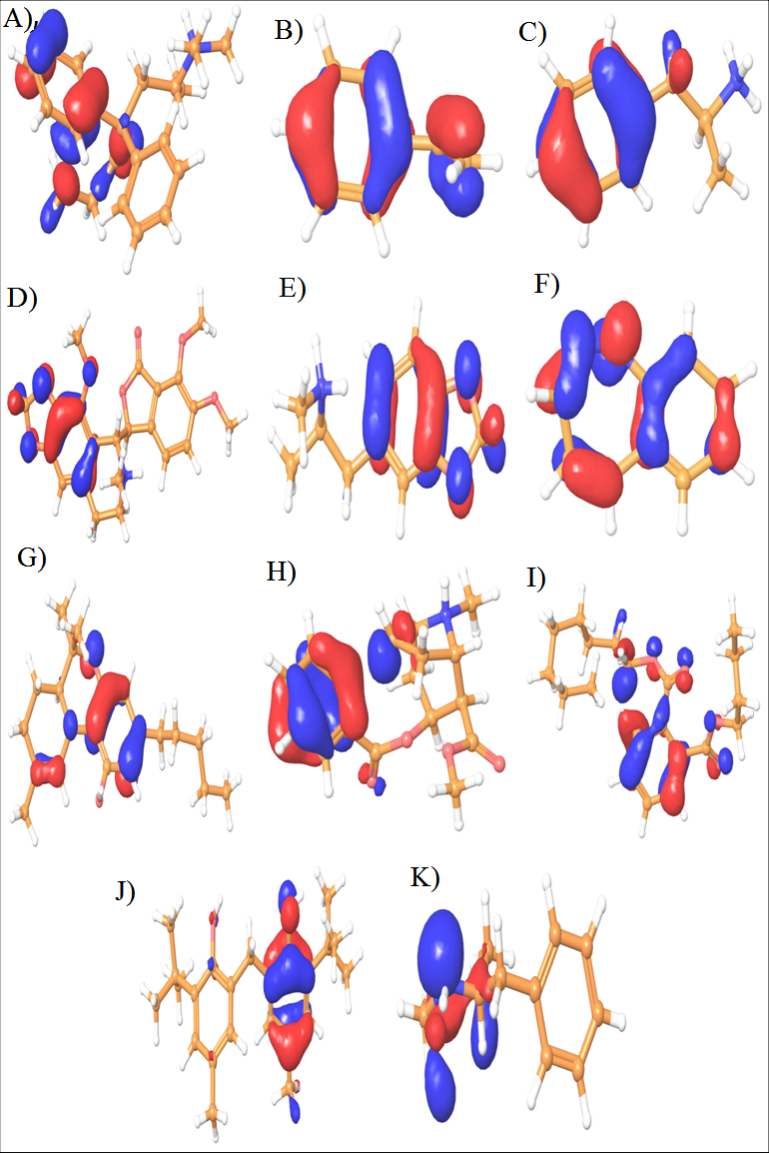
3-D counter map analysis of Highest Occupied Molecular Orbital (HOMO) for eleven illicit drugs and bomb compounds. The eleven compounds are A) Methadone, B) Binder styrene Butadiene C) Amphetamine D) Narcotine E) Ectasy F) Benzodiazepine G) Dronobinal Cannabis H) Crack Cocaine I) Plasticizer J) N-Phenyl-2-Napthalamine and K) Methamphetamine.

**Figure 9:**
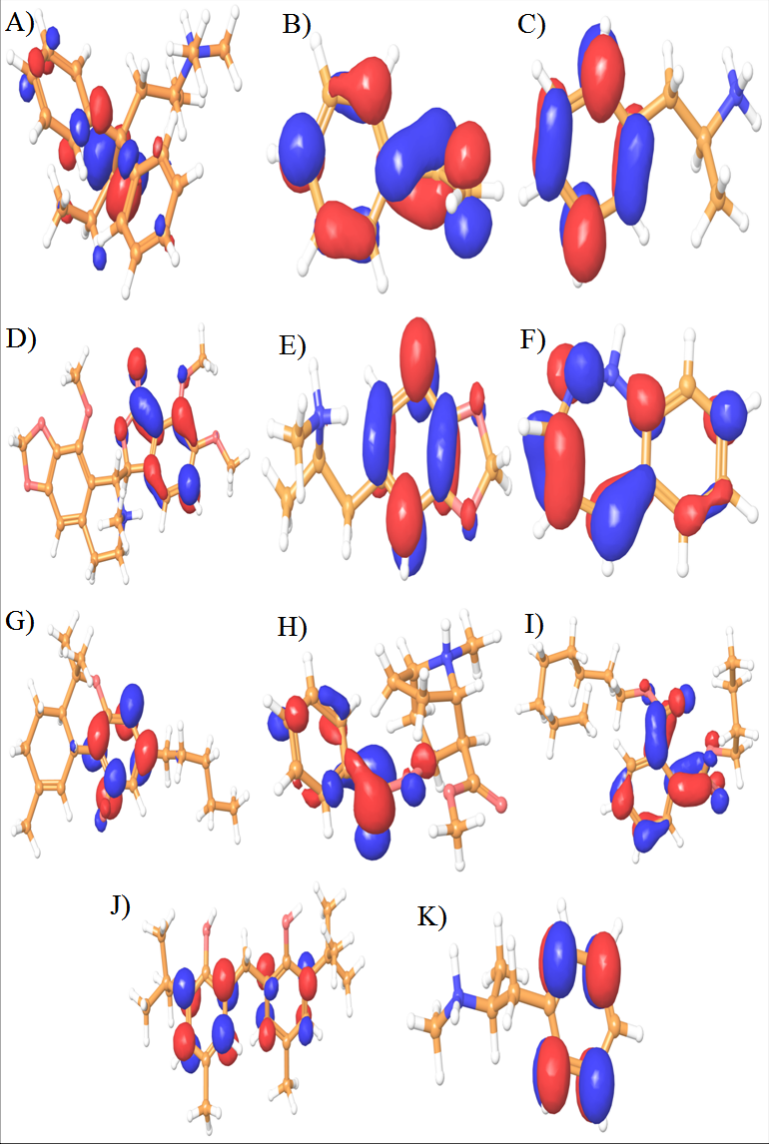
3-D counter map analysis of Lowest Unoccupied Molecular Orbital (LUMO) for eleven illicit drugs drugs and bomb compounds. The eleven compounds are A) Methadone, B) Binder styrene Butadiene C) Amphetamine D) Narcotine E) Ectasy F) Benzodiazepine G) Dronobinal Cannabis H) Crack Cocaine I) Plasticizer J) N-Phenyl-2-Napthalamine and K) Methamphetamine.

DFT study implies the frontier orbital energy including Highest Occupied Molecular Orbital (HOMO) and Lowest Unoccupied Molecular Orbital (LUMO) to understand the electron transfer feature of eleven compounds. The electron donor/acceptor properties of the molecules were indicated by the distribution of frontier molecular orbital's that illustrates the favorable sites for nucleophilic (HOMO) and electrophilic (LUMO) attack during charge transfer reaction. The HOMO and LUMO energy gap defines the internal charge transfer interaction among the compounds. Lowering gap energy implied the less stability with high chemical reaction of the compounds. The compounds Amphetamine, Ecstasy, N-Phenyl-2-Napthalamine, Benzodiazepine, Dronobinal Cannabis, Crack Cocaine, Methamphetamine, and narcotine consist of one or more aromatic rings, lipophilic and aliphatic groups in the chemical moiety. Therefore, it is important for the discrimination of honeybee OBPs for binding and recognition of the olfactory system [31]. HOMO-LUMO regions are localized in aromatic, lipophilic, aliphatic, amine (-NH3) and hydroxyl groups (-OH) of N-Phenyl-2-Napthalamine, Benzodiazepine, Crack Cocaine, Methamphetamine and narcotine compounds form H-bond, pi-pi stacking and Cation-pi stacks interaction interactions with Leu, Lys, Val, Asp and Asn amino acids for chemosensory signaling reaction for honey bees olfactory system (Venthur et al. 2014). It has been reported that aromatic, lipophilic and aliphatic group of ligand molecules are important features for binding affinity and chemosensory signaling in OBPs. This HOMO-LUMO energy gap is the improved indicator for electron transport mechanism in the molecule. All the compounds have low HOMO-LUMO energy gaps shown in (Table 4). This interaction may favor for the recognition and identification of illicit drugs and bomb compounds. The stability of the reactions was identified using HOMO-LUMO gap that renders that, all the compounds may have more reactive with less band gap for the biological reactions. The HOMO-LUMO regions of eleven illicit drugs and bomb compounds were shown in (Figure 8 and Figure 9).

## Conclusion

Analysis of OBP across distant phylogeny is of interest is the development of biosensor application. We report the binding of 19 compounds with 43 OBPs from distant phylogeny using modeling and docking analysis. Honeybees are important pollinator and it is used in the several ways, such as medical, agricultural, etc., and due to their learning power, it is used to detect bombs and some illicit drugs compounds. This extensive in silico approach is preliminary work to understand and explain the detecting mechanism of illicit drugs and bomb compounds, therefore we can overcome the solution by taking experimental evidence so far done. The phylogenetic tree analysis of OBPs from Apis Millefera and Apiscerana explains that proteins were highly similar in nature. Until now, Apis Millefera used for the detection and training illicit drugs, rather from this current study, Apis Cerana can be used to treat such training. The electrostatic interactions of OBPs are highly influenced the compound to bind and prefer the reaction to identify the location of sources. Consequently, the docking protocol had been helped to identify the binding preference and interaction of the compound to understand the biological function of proteins. Based on the docking, the binding preference and docking score of the complexes can be used to train or molecular level biosensor application to detect the illicit drugs and bomb compounds using honeybee. Also, the electronic feature of the compounds can be used to understand the chemical reactions to stimulate the memory power of OBPs. HOMO-LUMO regions and their energy gap define the chemical reaction of compounds with OBPs leads to understand the stimulating mechanism for finding the illicit drugs and bomb. Understanding the molecular interaction and chemical reaction of the compound may help to understand the fundamental of sensing reactions. Moreover, concentrating on these proteins at the molecular level will pave the potential role in the detection of illegal drugs and bomb compounds using honeybee.

## Conflict of interest

The authors declared that there are no conflicts of interest.

## Figures and Tables

**Table 1:** Nineteen illicit drugs and bomb compounds collected from PubChem database.

S .no	Compound name	PubChem ID
1	Rdx	8490
2	Binder styrene Butadiene	62697
3	Trinitrotoluene	69044
4	Semte	56841778
5	Ecstasy	1615
6	Methadone	4095
7	Narcotic	4544
8	Crack cocaine	5760
9	Amphetamine	5826
10	Petn	6518
11	N-phenyl-2-Napthalamine	8398
12	Methamphetamine	10836
13	Dronobinal cannabis	16078
14	Plasticizer	66540
15	Benzodiazepine	134664
16	Crack Cocaine	446220
17	Tri-cyclic acetone peroxide	4380970
18	Heroine	5462328
19	Bath salt mdpv	20111961

**Table 2:** Docking score of 43 proteins with 19 Illicit drugs and bomb compounds.

PubChem ID	PROTEIN ID	Compound name	Docking score
66540	1TUJ	Plasticizer	-6.876
16078		Dronobinal cannabis	-6.65
5826	2H8V	Amphetamine	-6.824
1615		Ecstasy	-6.505
1615	3BJH	Ecstasy	-10.894
5760		Crack cocaine	-10.864
66540	3CYZ	Plasticizer	-10.142
5760		Crack cocaine	-9.491
7658	3D73	Phenylethyl butanoate	-9.209
6054		Phenethyl alcohosl	-7.901
5760	3D75	Crack cocaine	-10.445
8398		N-phenyl-2-napthylamine	-10.052
8398	3FE6	N-phenyl-2-napthylamine	-10.807
5760		Crack cocaine	-10.311
66540	3R72	Plasticizer	-8.604
8398		N-phenyl-2-napthylamine	-7.925
134664	3RZS	Benzodiazepine	-8.98
62697		Binder styrene butadiene	-6.34
134664	3SOA	Benzodiazepine	-8.113
5826		Amphetamine	-5.992
5760	3D75	Crack cocaine	-10.445
5760		Crack cocaine	-9.837
16078	3D78	Dronobinal cannabis	-11.667
66540		Plasticizer	-10.861
5826	AOAOA7RDX8 (OBP1)	Amphetamine	-6.477
62697		Binder styrene butadiene	-6.329
134664	AOAOKOPX79 (OBP2)	Benzodiazepine	-6.509
62697		Binder styrene butadiene	-6.224
134664	AOAOKOPX82 (OBP3)	Benzodiazepine	-8.851
5826		Amphetamine	-6.173
5760	AOAOKOPXH2 (OBP4)	Crack cocaine	-4.65
16078		Dronobinal cannabis	-4.645
4095	AOAOKOPXY3 (OBP5)	Methadone	-10.493
66540		Plasticizer	-9.186
134664	AOAOU2SP42 (OBP6)	Benzodiazepine	6.891
5826		Amphetamine	-6.164
134664	AOAOU2SQWO (OBP7)	Benzodiazepine	-8.913
62697		Binder styrene butadiene	-6.22
16078	AOAOU2UB85 (OBP8)	Dronobinal cannabis	-3.588
10836		Methamphetamine	-3.174
5760	H6VYYO	Crack cocaine	-11.537
4095	(OBP9)	Methadone	-10.642
5760	H6VYY1	Crack cocaine	-11.758
4095	(OBP10)	Methadone	-11.712
134664	V9IM79	Benzodiazepine	-6.503
62697	(OBP11)	Binder styrene butadiene	-6.208
5826	AOAOU2SR55 (OBP12)	Amphetamine	-4.334
66540		Plasticizer	-4.174
66540	S5CRW7	Plasticizer	-10.322
16078	(OBP13)	Dronobinal cannabis	-9.993
16078	Q8WRW3 (OBP14)	Dronobinal cannabis	-8.409
66540		Plasticizer	-7.879
5760	Q8WRW4 (OBP15)	Crack cocaine	-6.5
5760		Crack cocaine	-6.294
8398	Q8WRW5 (OBP16)	N-phenyl-2-napthylamine Plasticizer	-12.253
66540		-10.559
8398	Q8WRW5	N-phenyl-2-napthylamine Plasticizer	-12.087
66540	(OBP17)		-11.274
5760	Q8WRW6 (OBP18)	Crack cocaine	-6.415
4095		Methadone	-6.356
62697	Q9U9J5	Binder styrenebutadiene	-5.639
1615	(OBP19)	Ecstasy	-2.584
5760	Q9U9J5	Crack cocaine	-7.549
5760	(OBP20)	Crack cocaine	-7.516
8398	Q9U9J6_ASP1 (OBP21)	N-phenyl-2-napthylamine Plasticizer	-12.087
66540		-11.274
5826	Q1W1D7	Amphetamine	-5.763
10836	(OBP22)	Methamphetamine	-5.015
1615	Q1W1D8	Ecstasy	-5.128
8398	(OBP23)	N-phenyl-2-napthylamine	-4.655
16078	Q1W1E0	Dronobinal cannabis	-7.604
1615	(OBP24)	Ecstasy	-6.752
5760	Q5VK57	Crack cocaine	-7.195
5760	(OBP25)	Crack cocaine	-7.136
4095	V91HTO	Methadone	-3.336
4544	(OBP26)	Narcotine	-3.08
8398	MODEL 2 (OBP27)	N-phenyl-2-Napthylamine	-5.308
5826		amphetamine	-5.173
8398	Q8WRW2 (OBP28)	N-phenyl-2-Napthylamine	-7.749
6654		0plasticizer	-7.082
8398	V9VFX4	N-phenyl-2-Napthylamine	-5.308
5826	(OBP29)	Amphetamine	-5.173
5760	V91F66	Crack cocaine	-8.578
1615	(OBP30)	Ecstasy	-7.867
1615	X2GEC7	Ecstasy	-6.243
5826	(OBP31)	Amphetamine	-6.079

**Table 3:** Sequence information and source of organism of 43 OBPs.

S.no	Protein Id	Sequence Id	Source of organism
1	1TUJ	Q9U9J5	Apis mellifera
2	2H8V	Q8WRW5	Apis mellifera
3	3BJH	Q8WRW5	Apis mellifera
4	3CYZ	Q9U9J6	Apis mellifera
5	3D73	Q9U9J6	Apis mellifera
6	3D75	Q9U9J6	Apis mellifera
7	3FE6	Q9U9J6	Apis mellifera
8	3R72	Q8WRW2	Apis mellifera
9	3RZS	Q1W640	Apis mellifera
10	3SOA	Q9UQM7	Apis mellifera
11	3D75	Q9U9J6	Apis mellifera
12	3D78	Q9U9J6	Apis mellifera
13	OBP 17	A0A0A7RDX8	Apis cerana cerana
14	OBP 21	A0A0K0PX79	Apis cerana cerana
15	OBP 14	A0A0K0PX82	Apis cerana cerana
16	OBP 12	A0A0K0PXH2	Apis cerana cerana
17	OBP 15	A0A0U2SP42	Apis cerana cerana
18	OBP 14	A0A0U2SQW0	Apis cerana cerana
19	OBP 12	A0A0U2UB85	Apis cerana cerana
20	OBP 1	H6VYY0	Apis cerana cerana
21	OBP 1	H6VYY1	Apis cerana cerana
22	OBP 21	V9IM79	Apis cerana cerana
23	OBP 13	A0A0U2SR55	Apis cerana cerana
24	OBP OBP11	S5CRW7	Apis cerana cerana
25	OBP ASP6	Q8WRW3	Apis mellifera
26	OBP ASP4	Q8WRW4	Apis mellifera
27	OBP ASP1	Q8WRW5	Apis mellifera
28	OBP ASP1	Q8WRW5	Apis mellifera
29	OBP ASP4	Q8WRW6	Apis mellifera
30	OBP ASP2	Q9U9J5	Apis mellifera
31	OBP ASP2	Q9U9J5	Apis mellifera
32	PBPASP1	Q9U9J6	Apis mellifera
33	OBP ASP1	Q1W1D7	Apis cerana cerana
34	OBP ASP3	Q1W1D8	Apis cerana cerana
35	OBP ASP2	Q1W1E0	Apis cerana cerana
36	OBP 24	Q1WI24	Apis cerana cerana
37	OBP ASP4	Q5VK57	Apis cerana cerana
38	OBP 23	Q6VK37	Apis cerana cerana
39	OBP 27	Q9WY56	Homo sapiens
40	OBP ASP5	Q8WRW2	Apis mellifera
41	OBP 3	V9VFX4	Apis cerana
42	OBP 10	V9IF66	Apis cerana
43	OBP 3	X2GEC7	Apis cerana
OBP - Odorant Binding Protein; PBP - Pheromone-binding protein			

**Table 4:** DFT analysis result for the top eleven illicit drugs and bomb compounds.

Compounds	HOMO (eV)	LUMO (eV)	EHOMO-ELUMO (eV)	Solv.Energy (kcal/mol)
Crack Cocaine	-0.24	-0.05	-0.18	-0.05
Plasticizer	-0.26	-0.07	-0.19	-0.05
N-Phenyl-2-Napthalamine	-0.21	-0.01	-0.2	-0.05
Dronobinal Cannabis	-0.21	0	-0.21	-0.05
Benzodiazepine	-0.18	-0.07	-0.11	-0.05
Binder styrene Butadiene	-0.24	-0.01	-0.22	-0.05
Methadone	-0.23	-0.04	-0.18	-0.05
Narcotine	-0.21	-0.06	-0.14	-0.05
Methamphetamine	-0.23	0	-0.22	-0.05
Ecstasy	-0.21	-0.01	-0.19	-0.05
Amphetamine	-0.24	-0.01	-0.23	-0.05
